# Women and Their Victims

**Published:** 1888-11-10

**Authors:** 


### WOMEN AND THEIR VICTIMS.

I must say I have been rather exercised in mind since reading the article with the above heading in The Hospital, and although I do not myself follow the fashion of adorning my garments with beautiful birds, I cannot see why the custom should be called such hard names, nor the ladies who follow it considered such utterly cruel and heartless beings, lost to all feelings of humanity, as the writer evidently thinks them. I do not see that there is any more harm in using animals as ornaments than in using them as food, indeed the number used in the former capacity must be infinitesimal compared with that used in the latter, and I do not suppose the pang of death can be any greater to a bird that is destined to adorn a bonnet than to one that is fated to appear in a pie. We might as well spend our lives in bewailing the wholesale destruction to small animal life which we are told takes place every time a whale makes a meal, as to spend it in grieving over those quantities of pretty little foreign birds which are at present made a prey of to satisfy a passing demand of fashion, instead of being left in peace to meet their fate at the hands of their natural enemies—serpents, or other wild animals probably.

A Woman Who Does Not Wear Birds.

[Our correspondent has missed the main point of the indictment. Birds are taken in the nesting season for ornaments, and the young are left to die of slow starvation. No sportsman shoots birds for food during that season.—Ed. *T. H.*]

